# Guanidino compounds with native GABA(A) δ receptor selectivity: a tale of homeostatic compensation in δ-KO mice

**DOI:** 10.1186/s12868-025-00987-z

**Published:** 2025-12-11

**Authors:** Pratap Meera, Mikko Uusi-Oukari, Martin Wallner, Gerald S. Lipshutz

**Affiliations:** 1 Department of Neurobiology, University of California, Los Angeles, CA 90095 USA; 2 Integrative Physiology and Pharmacology, Institute of Biomedicine, University of Turku, Turku, FIN-20014 Finland; 3 Department of Surgery, University of California, 635 Charles E. Young Dr. S., NRB 379D, Los Angeles, CA 90095 USA; 4 Molecular & Medical Pharmacology, University of California, Los Angeles, CA 90095 USA; 5 Intellectual and Developmental Disabilities Research Center, University of California, Los Angeles, CA 90095 USA; 6 Semel Institute for Neuroscience, University of California, Los Angeles, CA 90095 USA

**Keywords:** GABA_A_ receptor, GABA-mimetics, Extrasynaptic GABA_A_ receptors, Guanidinoacetate, Guanidinopropionate

## Abstract

Altered GABAergic transmission has been implicated in the neurological symptoms of metabolic disorders associated with guanidino compound (GC) accumulation. Building on previous findings that selected GCs act as direct orthosteric GABA_A_​ receptor (GABAR) agonists, we now asked whether these GCs act preferentially on high-affinity extrasynaptic δ subunit-containing receptors (δ-GABAs). Using whole-cell patch clamp recordings from mouse cerebellar granule cells (CGCs) in brain slices of wild-type and δ-subunit knockout (δ-KO) mice, together with 5 nM [³H]muscimol displacement assays on WT and δ-KO forebrains, we compared the actions of four structurally GABA-like GCs — guanidinoacetate (GAA), β-guanidinopropionate (β-GPA), guanidinoethanesulfonate (GES), and γ-guanidinobutyrate (γ-GBA). These compounds activated CGC GABARs in cumulative concentration-response curves and displaced the highly δ-GABAR-selective ligand [^3^H]muscimol suggesting δ-GABAR-selective orthosteric agonist actions. In δ-KO forebrains, total [³H]muscimol binding was reduced by ~ 50%, confirming the loss high-agonist-affinity but low-abundance (~ 5% of total forebrain GABARs) δ-GABARs. δ-KO CGCs showed markedly reduced agonist sensitivity, with EC₅₀ values (µM, WT/δ-KO): GABA (2/6) < β-GPA (3/8) < GAA (4/14) < GES (32/72) < γ-GBA (44/219). The modest loss of agonist sensitivity for GABA and the four GABA-mimetic GCs in δ-KO CGCs is consistent with compensatory upregulation of non-δ extrasynaptic GABARs containing only α and β subunits, as previously described (Tretter et al., JBC 2001), explaining the preserved tonic inhibition in δ-KO neurons. Our findings demonstrate that GABA-mimetic GCs preferentially target δ-GABARs and suggest that homeostatic compensation by αβ-type GABARs is a key adaptive mechanism maintaining inhibitory tone in δ-KO CGC neurons.

## Introduction

Altered GABAergic transmission has long been implicated in the neurological symptoms associated with metabolic disorders involving the urea and creatine metabolic pathways [1, 2]. Such disorders, including acute and chronic liver and kidney failure [3–6] and congenital enzyme deficiency disorders are often accompanied by neurotoxicity despite the protective function of the blood-brain barrier. The accumulation of uremic and hepatic toxins primarily affects the central nervous system (CNS), causing cognitive impairment, seizures, and other neurological manifestations. Yet, despite decades of research, the identity of these toxins, their molecular targets and mechanisms remain largely elusive. Among the suspected mediators are guanidino compounds, although hyperargininemia and hyperammonemia are also implicated in neurotoxicity [2, 7–10].

**Fig. 1:**
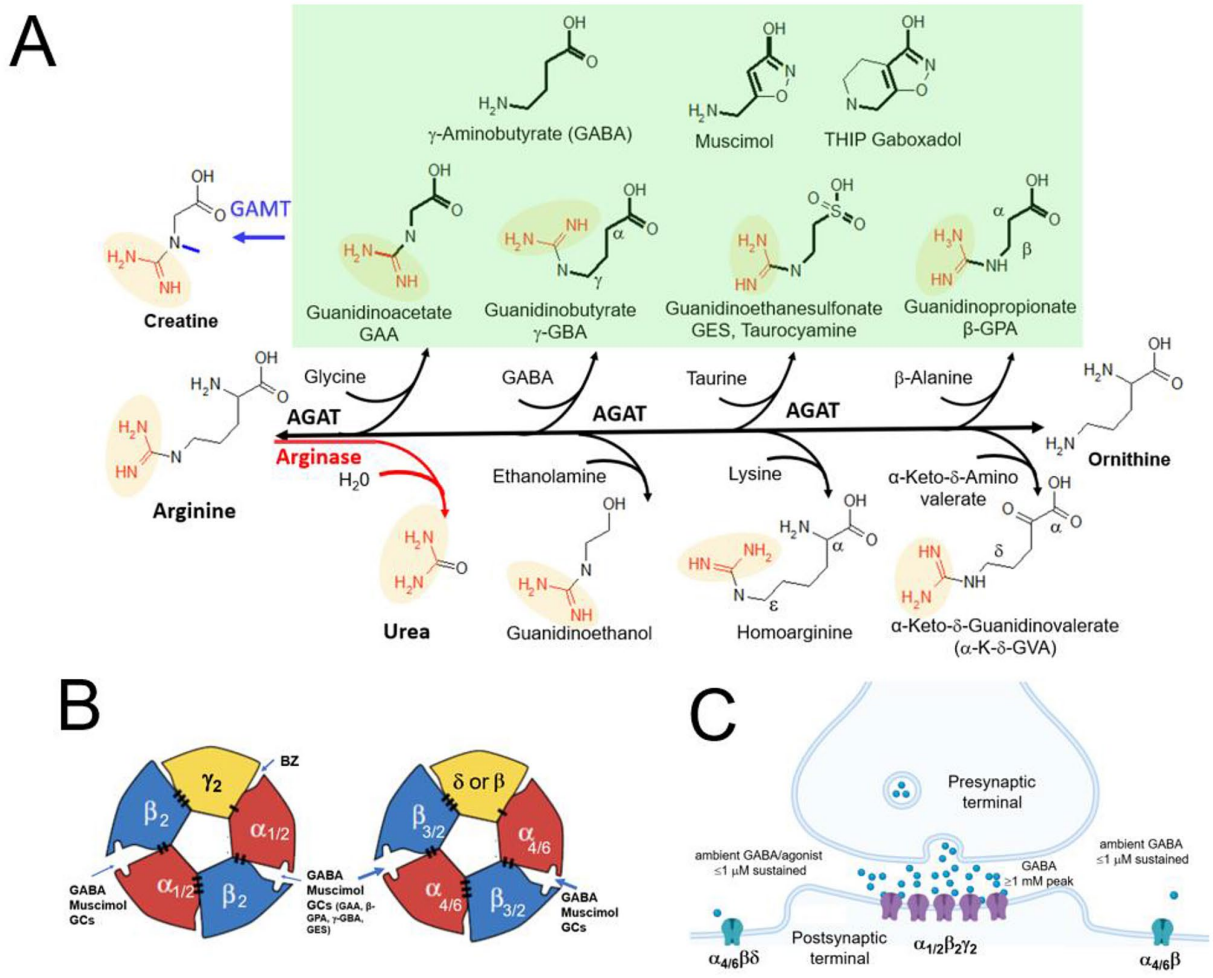
Structures and biosynthesis of GABA-like guanidino compounds (**A**) Biosynthetic pathways involving the enzymes arginase, AGAT and GAMT. The GABA backbone structure is in bold, and the amidino group is in red and shaded in yellow. GAA, γ-GBA, GES and β-GPA are grouped with GABA (and the conformationally restricted GABA analogs THIP and muscimol) and shaded in green, all of which activate native GABARs and displace [^3^H]muscimol from mouse brain native (WT and δ-KO) GABARs. Based on structural requirements, urea, guanidinoethanol, homoarginine and α-K-δ-GVA are unlikely to bind at reasonable concentrations to the GABA/muscimol site; we confirmed absence of detectable CGC GABAR activity at concentrations up to 1 mM for arginine, creatine and α-K-δ-GVA as well as absence of [^3^H]muscimol (5 nM) displacement at concentrations up to 0.3 mM in mouse forebrain. Muscimol and THIP are conformationally restricted high affinity orthosteric GABA ligands, with particularly high affinity for extrasynaptic δ subunit-containing GABARs. (**B**) Subunit arrangement of GABA_A_R showing two orthosteric agonist at β/α subunit interfaces, with the classical benzodiazepine (BZ) binding site at the α/γ2 subunit interface. In δ-containing GABARs, the δ subunit replaces the γ2 subunit. In GABARs composed of only α and β subunits, there is a third β subunit at the same position (marked in yellow). Agonist/GABA binding triggers conformational changes that open the central Cl^−^ permeable pore. (**C**) Dichotomy between synaptic (low affinity) and high affinity extrasynaptic GABA_A_ receptors. Most classic synaptic GABA receptors are composed of α1, β2 and γ2 subunits and mediate inhibitory synaptic transmission triggered by brief (milliseconds) bursts of high (≥1 mM) GABA concentrations in the synaptic cleft. In marked contrast, extrasynaptic GABARs (composed of α4/6βδ and/or αβ subunits) sense low ambient GABA concentrations ≤ 1 µM

Human genetic disorders and their animal models provide specific insight into the molecular mechanism of these metabolic encephalopathies, since congenital enzyme defects generally lead to the accumulation of specific metabolites, with hyperexcitability and epilepsy a frequent phenotype [11]. A notable example is guanidinoacetate (GAA) in N-methyltransferase (GAMT) deficiency, in which the final step in creatine synthesis is blocked (see Fig. 1A, top left). GAMT deficiency leads to a dramatic increase in guanidinoacetate (GAA), in both brain and the periphery while reducing creatine [12]. The neurological symptoms of GAMT deficiency appear to result mainly from GAA accumulation rather than from creatine deficiency, as even prolonged high-dose dietary creatine supplementation restores brain creatine with only limited effect on the neurological phenotype [13].

The creatine precursor GAA is formed by arginine: glycine amidinotransferase (AGAT) which transfers an amidino group from arginine (its rate-limiting substrate) to glycine. Due to its broad substrate specificity, AGAT can also generate other guanidino compounds, including β-guanidinopropionic acid (β-GPA), γ-guanidinobutyric acid (γ-GBA) and guanidinoethanesulfonic acid (GES) - from their precursor amino acids β-alanine, GABA and taurine, respectively (see Fig. 1A) [14, 15]. Under conditions such as arginase deficiency, where arginine accumulates, mass-action effects might drive overproduction of GAA and related GCs see Fig. 1 [16]. Conversely, γ-GBA, β-GPA and GES (but not GAA) can be hydrolyzed by a guanidino acid hydrolase, which might have evolved to detoxify these potentially harmful GCs [15]. Because GABA and GABA mimetic guanidino compounds are highly polar, they do not cross the blood-brain barrier in the absence of active transport mechanisms. Therefore, endogenous brain synthesis of GCs by the enzyme AGAT (Fig. 1) may be critical for their brain accumulation. Indeed, GAA activates GABARs at concentrations matching those found in the CSF in GAMT deficiency, implicating GAA agonist action in the associated neuropathology [1, 17].

β-GPA is a potent creatine transporter blocker [18, 19], competing with creatine for transport with an EC_50_ of ~ 13 µM in recombinant (HEK) cell expression [20]. This inhibition reduces intracellular ATP, which might underlie β-GPA’s anti-hyperglycemic and exercise mimetic actions [21, 22]. Given the high ATP demand of cancer cells, β-GPA is currently under investigation as a potential cancer therapeutic [23]. GES has been shown to be a taurine transporter substrate with a K_m_ of ~ 150 µM. Oral GES administration has been used to study the effects of (peripheral) taurine depletion [24].

Mammalian GABARs are heteropentameric complexes, with classical synaptic GABARs comprising the majority of all brain GABARs [25]. Synaptic GABARs most frequently contain two α1 subunits, two β2 subunits and one γ2 subunit arranged in a pseudo-symmetric α1β2α1β2γ2 configuration [26] (see Fig. 1B). Incorporation of a γ2 subunit is necessary for (post)synaptic γ2-GABARs localization and confers relatively low GABA sensitivity, such that γ2-GABAs are primarily activated by brief bursts of high (mM) GABA concentrations of GABA released into the synaptic cleft [27, 28] (Fig. 1C). In contrast, extrasynaptic GABARs lack the γ2 subunit, which is replaced by the δ subunit – or by a third β subunit [29]. Additionally, δ-GABARs contain either α4 (forebrain) or α6 in cerebellar granule cells [30, 31] (Fig. 1C). The absence of γ2 results in GABARs with markedly higher GABA/agonist sensitivity [32], which is further increased by δ subunit incorporation [33, 34].

Unlike fast-desensitizing synaptic γ2-GABARs, extrasynaptic GABARs exhibit sustained activation in the continuous presence of low (≤1 µM) ambient GABA. Their high GABA/agonist sensitivity and lack - or very slow and incomplete - desensitization enable them to mediate a constant (tonic) form of inhibition. This sustained activity of extrasynaptic GABARs more than compensates for their low abundance, with α4βδ-GABARs constituting ~ 5% of total GABAR in the mouse forebrain [25] and ~ 20% α6βδ GABARs in the cerebellum [31]. Thus despite their low abundance, the combination of high GABA/agonist sensitivity combined with persistent activity renders extrasynaptic GABARs critical regulators of neuronal excitability [35] - notwithstanding the absence of obvious hyperexcitability in δ-KO mice, presumable due to homeostatic compensatory plasticity.

**Fig. 2:**
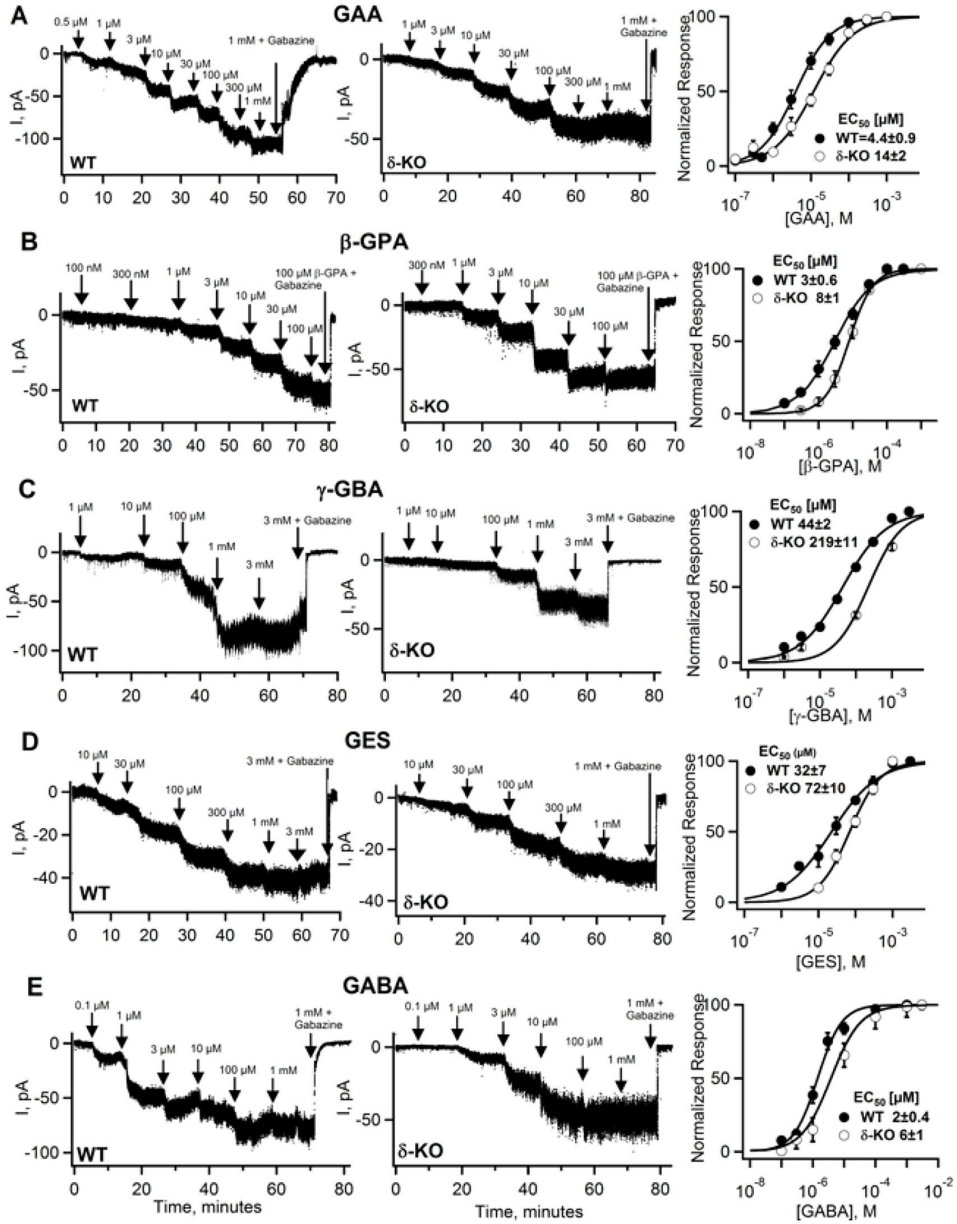
*The structural GABA analogs GAA*,* γ-GBA*,* β-GPA*,* GES*,* like GABA*,* evoke GABA receptor currents in mouse cerebellar granule cells (CGC) from WT and δ-KO mice.* Whole-cell patch clamp recordings of CGCs at a holding potential of -70 mV in the presence of the glutamate receptor blocker DNQX (10 µM) with action potentials blocked by 0.3 µM TTX. Currents in WT and δ-KO CGCs were evoked by perfusion of increasing concentrations (indicated by arrows) of (**A**) GAA, (**B**) β-GPA, (**C**) γ-GBA, (**D**) GES and (**E**) GABA for 5–10 min, until the currents reach a new steady state. After application of a saturating agonist concentration, responses were blocked by the GABAR-specific antagonist gabazine (SR95531) to confirm mediation by CGC GABARs. Concentration-response curves (right panels) were generated by fitting current amplitudes with the Hill equation to determine EC_50_ values and Hill slope coefficients. Statistical comparisons between WT and δ-KOs currents were performed using Student’s t-test with EC_50_ values determined by fitting each individual experiment and showed a moderate yet significant loss of agonist potency in δ-KO CGCs. For β-GPA the curve was significantly shallower in WT when compared to δ-KO CGCs (*p* = 0.003) and only γ-GBA showed significantly reduced current amplitude in the δ-KO (*p* = 0.017) (see Table 1 for data and statistics)

Here we demonstrate and confirm [17] that the GABA structural analogs GAA, β-GPA, γ-GBA, and GES are agonists on native GABARs in WT and less potently also in δ-KO CGCs. (Fig. 2A).

Agonist actions were confirmed by displacement of the orthosteric radioligand [³H]muscimol at 5 nM, a highly δ-GABAR–selective concentration [36]. In WT membranes GAA, β-GPA, γ-GBA, and GES displaced around 50% δ-GABAR specific [^3^H]muscimol binding that is lacking in the δ-KO forebrain, with potencies comparable to those observed with electrophysiology, consistent with the notion that this reflects δ-GABARs selectivity. Interestingly, [^3^H]muscimol displacement by GAA, β-GPA, GES and GABA of the remaining 50% (likely artefactual) high-affinity [^3^H]muscimol binding in δ-KO brains suggests comparable potency to this poorly characterized fraction of high affinity [^3^H]muscimol binding [36]. A notable exception is γ-GBA, with loss of displacement potency and reduced maximal currents in δ-KO CGCs. While δ subunit deficiency significantly reduces agonist potency, the effect is unexpectedly modest and surprisingly no reduction (with the exception of γ-GBA) in maximal current amplitude, for not only GAA, β-GPA, GES, but also GABA itself. Given that synaptic receptors are expected to be largely desensitized, this likely reflects an under-appreciated compensatory expression of fairly agonist sensitive “binary” α6β extrasynaptic receptors [31], to replace extrasynaptic α6βδ GABARs in δ-KO cerebellar granule cells (CGCs).

## Materials and methods

### Animal (mouse) methods

Animal care and experimental procedures were as described [17, 37] and conducted according to the guidelines of the UCLA Chancellor’s Animal Care and Use Committee (ARC) (Animal Welfare Assurance number A3196-01 and animal protocol ARC-2019-032) and kept according to the National Institutes of Health guidelines with temperature and humidity control. We used C57BL/6 wildtype mice and δ-KO mice (a gift from Istvan Mody at UCLA) on the C57BL/6 background (both sexes, 6–16 weeks of age, total about 115 mice). The mice were housed in conventional cages in 12:12 h light: dark cycle in groups of 4–5 mice per cage having *ad libitum* access to rodent lab chow (PicoLab Rodent Diet 20, #5053) and water. Brains were removed after brief isoflurane anesthesia, the cerebellum was used for preparing cerebellar brain slices, and forebrains were stored at -80 °C and shipped on dry ice for [^3^H]muscimol displacement experiments. Wild type C57Bl/6 mice were bred within our mouse colony as were δ-KO mice.

### Brain slice preparation and electrophysiology

Slices were prepared using standard techniques: Cerebella were removed from the cranium, submerged in cold (< 4 °C) artificial cerebrospinal fluid (aCSF), and ~ 290 µM thick slices were cut with a (Leica VT-1000s, Deerfield, IL, USA) vibratome. The slicing solution contained (in mM): 85 NaCl, 0.5 CaCl_2,_ 4 MgCl_2_, 2.5 KCl, 1.25 NaH_2_PO_4_, 75 sucrose, 24 NaHCO_3_, 25 glucose. Slices were oxygenated with 95% O_2_ and 5% CO_2_ in artificial cerebrospinal fluid (aCSF) for storage and electrophysiological recordings containing (in mM): 119 NaCl, 2.5 KCl, 2.5 CaCl_2_, 1.3 MgCl_2_, 1 NaH_2_PO_4,_ 26 NaHCO_3_ and 11 glucose. Whole cell pipette solution consisted of (in mM): 100 KCl, 5 NaCl, 4 MgCl_2_, 4 ATP and 0.4 GTP, 40 HEPES adjusted to pH 7.4 with KOH. All salts/chemicals were obtained from Millipore-Sigma (Saint Louis, MO, USA). Cerebellar granule cells (CGCs) were visualized using an infrared-DIC enhancement equipped upright microscope (Zeiss, White Plains, NY) and whole cell CGC recordings performed with a Multiclamp 700B amplifier (Axon Instruments, Inc., Foster City, CA, USA) at -70 mV at room temperature (22–24 °C). Action potentials and glutamate receptor-mediated transmission were blocked by 0.3 µM TTX and 10 µM DNQX respectively.

### Reagents

GABA, γ-GBA and β-GPA were purchased from Sigma-Aldrich (St. Louis, MO, USA). Guanidinoacetic acid (GAA) was from Pfaltz & Bauer (Waterbury, CT, USA) and GES (taurocyamine) was purchased from Cayman Chemical (Ann Arbor, MI, USA). [Methylene-^3^H]muscimol (22 Ci/mmol) was obtained from PerkinElmer Life and Analytical Sciences (Boston, MA, USA). GABA, GAA, β-GPA, GES, γ-GBA were prepared as 100 mM or 10 mM stock solutions either in aCSF for electrophysiology, or in assay buffer for [^3^H]muscimol displacement studies. Gabazine (SR95531), TTX, and DNQX were obtained from Tocris Bioscience, Inc. (Minneapolis, MN, USA) or from Hello Bio (Princeton, NJ, USA).

### [^3^H]Muscimol displacement experiments

Brain (*sans* cerebellum) membranes from WT and δ-KO mice were prepared as described [38] and stored frozen at -70 °C. After thawing, membranes were washed once in assay buffer (50 mM Tris-HCl, pH 7.4) and incubated for 40 min with 5 nM [^3^H]muscimol in assay buffer in a total volume of 300 µl at room temperature. Non-specific binding was determined by adding 100 µM GABA. After incubation, membranes were collected by filtration through Whatman GF/B filters (Whatman International Ltd., Maidstone, UK) with a Model M-24 Brandel Cell Harvester (Gaithersburg, MD, USA) followed by two quick washes with 4–5 ml of ice-cold assay buffer. Three milliliters of Optiphase HiSafe 3 scintillation fluid (Wallac, Turku, Finland) were added to air-dried filters and GABAR bound [^3^H]muscimol radioactivity determined in a Hidex 600 SL liquid scintillation counter (Hidex, Turku, Finland).

### Data analysis

GraphPad Prism (versions 7–10) software (GraphPad, San Diego, CA, USA), Microsoft Excel and Igor Pro were used for [^3^H]muscimol displacement and electrophysiology data analysis. Data are presented as mean ± standard error of mean (SEM) with statistical comparisons performed by Student’s t-test (unpaired, two-tailed) in Microsoft Excel and one-way ANOVA with Tukey’s multiple-comparison test in GraphPad Prism.

## Results

Because the guanidino compound GAA likely exerts its neurotoxicity through actions at GABARs [1, 37], we studied four structural GABA analogous GCs - GAA, γ-GBA, β-GPA, GES - for their ability to evoke GABAR-mediated currents in CGCs from both WT and δ-GABAR-deficient mice, as we suspected that GABA-mimetic actions are mediated primarily by high-agonist-affinity δ subunit-containing GABARs.

GABARs are frequently studied using recombinantly expressed receptors with defined subunit combinations. However recombinant systems have notable limitations. In particular, δ-GABARs are difficult to express functionally, often resulting in GABARs composed of only α and β subunits which do not fully reflect the high GABA, THIP and muscimol sensitivity observed in native δ-GABARs [34, 36, 39]. Furthermore, key modulatory proteins such as Neuroligin-2 [28], Shisa7 [40, 41] and TMEM132B, which are known to change pharmacological and functional properties [40, 42, 43] as well as endogenous bound neurosteroids [26] are likely missing in recombinant expression systems.

To circumvent these limitations, we examined native GABARs in CGCs from acutely prepared brain slices. CGCs, which constitute roughly 50% of all neurons in the mammalian brain, express well-characterized synaptic GABARs (mainly α1β2γ2 and α6βγ2 receptors) as well as extrasynaptic α6βδ GABARs. Notably, cerebellar extrasynaptic δ-GABARs comprise ~ 20% of total cerebellar GABARs [28, 31] – significantly higher than the ~ 5% of extrasynaptic α4βδ GABARs found in the forebrain [30]. This high abundance should make CGCs uniquely suited for studying the relative contributions of synaptic and extrasynaptic GABARs by comparing agonist-evoked responses to GABA and guanidino GABA mimetics in WT and δ-KO CGCs.

To measure the activation of native GABAR-mediated currents, we performed whole cell patch-clamp recordings from CGC using cumulative concentration-response experiments in which progressively higher agonist concentrations were applied. Under continuous agonist exposure, synaptic GABARs are expected to desensitize; thus evoked currents are primarily mediated by non-desensitizing extrasynaptic GABARs. As illustrated in Fig. 2, such slow perfusion conditions result in steady-state GABA currents that are arguably most relevant for GABA-mimetic compounds, which - like ambient GABA - are likely found in the extracellular space and detected by the high-affinity extracellular binding sites of extrasynaptic GABARs.

**Fig. 3:**
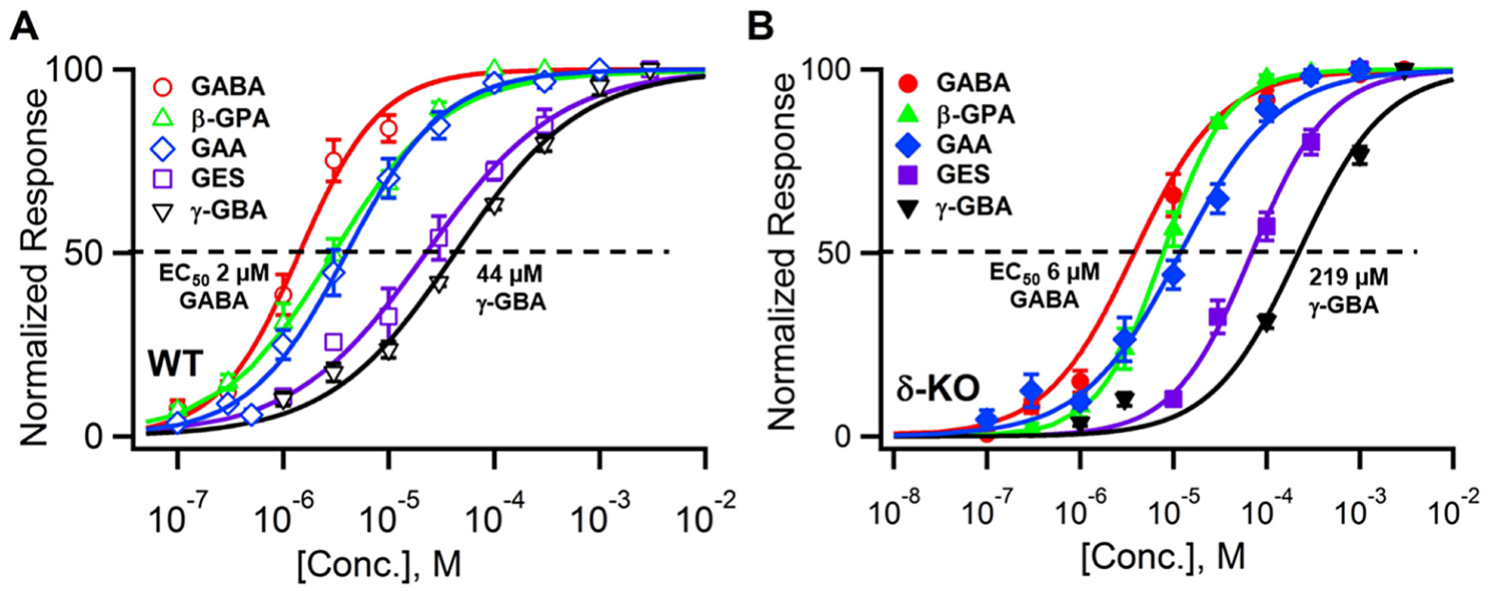
*Comparison of GAA*,* β-GPA*,* GES γ-GBA and GABA potency in WT* (**A**) *and δ-KO cerebellar granule cells (CGC)* (**B**). The EC_50_ values in WT CGCs range from 2 µM for GABA to 44 µM for γ-GBA, and from 6 µM for GABA to 219 µM for γ-GBA in δ-KO CGCs. See Table 1 for tabulation of number of experiments (n) and EC_50_ values. Error bars are standard error of mean (SEM). One-way ANOVA with Tukey’s multiple-comparison test indicated that most pairwise comparisons of EC_50_ values were highly significant (*p* < 0.001), except for GABA vs. GAA in WT (*p* = 0.03), and not significant (*p* > 0.05) for GABA vs. β-GPA, GAA vs. β-GPA in both WT and δ-KO, as well as GES vs. γ-GBA in WT CGC

Perfusion of increasing concentrations of GAA, β-GPA, γ-GBA and GES evoked currents in both WT and in δ-KO CGCs, similar to those observed with GABA, and these currents were completely blocked by the GABAR specific antagonist gabazine. Representative current traces for GAA, γ-GBA, GES, β-GPA and GABA in both WT and δ-KO CGCs are shown in Fig. 2A-E, and potency comparisons of electrophysiological recordings from WT and δ-KO CGCs are summarized in Figs. 3A, B. The EC_50_ values for half maximal activation, Hill slope, as well as the maximal currents evoked (with number of experiments in parentheses and variability expressed as ± SEM [standard error of mean]) are presented in Table 1.

**Table 1:** Analysis of EC_50_ and I_max_ values of GABA and the GABA-structural analogs GAA, β-GPA, γ-GBA, and GES of wild type and δ-KO CGCs GABA currents

	WT EC_50_ in µM (*n*)	δ-KO EC_50_ in µM (*n*)	pEC_50_ *p* value	WT slope	δ-KO slope	slope *p*-value	WT I_max_ pA	δ-KO I_max_ pA	I_max_ *p*-value
GAA	4.4 ± 0.9 (7)	14 ± 2 (7)	0.002 **	1.1 ± 0.16	0.94 ± 0.1	0.299	-60 ± 18	-50 ± 10	0.67
β-GPA	3.2 ± 0.6 (5)	8.2 ± 1.4 (6)	0.008 **	0.85 ± 0.1	1.3 ± 0.2	0.003**	-45 ± 9	-74 ± 19	0.18
γ-GBA	44 ± 2.3 (6)	219 ± 11 (5)	3 × 10^− 9^ **	0.81 ± 0.1	0.95 ± 0.1	0.17	-75 ± 12	-32 ± 7	0.017*
GES	32 ± 7 (5)	72 ± 10 (5)	0.017 *	0.89 ± 0.3	0.83 ± 0.2	0.8	-35 ± 7	-29 ± 6	0.48
GABA	1.8 ± 0.1(11)	6.1 ± 1 (8)	0.0002 **	1.5 ± 0.3	1.1 ± 0.1	0.3	-66 ± 5	-65 ± 18	0.92

EC_50_ values, hill slopes and maximal currents (I_max_) were determined from curve fits of individual experiments, with representative traces shown in Fig. 2. Also shown are maximal evoked currents (I_max_) at a saturating agonist concentration. Mean EC_50,_ hill slope and I_max_ values ± standard error of mean (SEM) are tabulated. Statistical comparisons between WT and δ-KOs currents (EC_50_ converted to pEC_50_s for t-test) were performed using student’s t-test (unpaired, two-tailed) in Microsoft excel with *p*>0.05 not significant, *p*<0.05 significant (*), and *p*<0.001 highly significant (**)

Among the tested compounds, GABA shows the highest potency (WT EC_50_ ~ 2 µM), followed closely by β-GPA and GAA (WT EC_50_ ~3 and 4 µM respectively) with GES and γ-GBA being less potent (WT EC_50_ ~32 and 44 µM respectively) (Fig. 3). As expected from the loss of high-affinity δ-GABARs, all compounds showed significantly decreased potency in δ-KO CGCs, with γ-GBA exhibiting the most pronounced loss of agonist-sensitivity in δ-KO CGCs (Fig. 2).

Maximal current amplitudes varied substantially across CGCs and, except for γ-GBA, did not differ significantly between WT and δ-KO CGCs. γ-GBA, was the only compound to evoke significantly reduced maximal currents (*p* = 0.017) in δ-KO CGCs (see Table 1).

By physically separating the cerebellum from the rest of the brain allowed us to investigate the two major δ-GABAR isoforms: cerebellar α6βδ receptors, examined by whole-cell recordings from cerebellar granule cells, and forebrain α4βδ receptors, analyzed in [³H]muscimol displacement assays. This approach allowed us to confirm that the tested guanidino compounds are GABA analogs and mimetics at the orthosteric GABA site in both major δ-GABAR subtypes [17, 37], and that effects are not unique to α6βδ-GABARs expressed in CGCs.

**Fig. 4:**
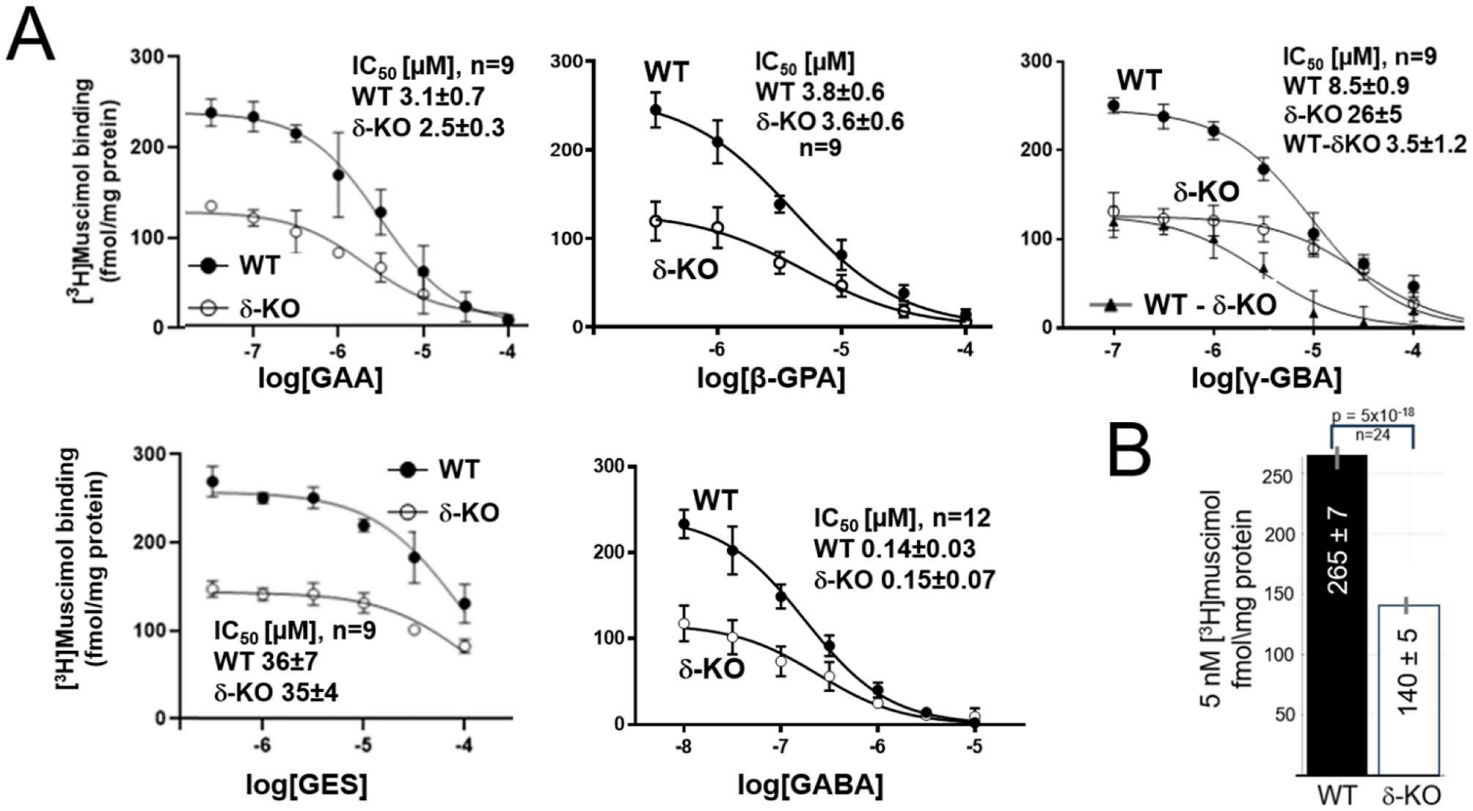
[^*3*^*H]muscimol displacement from WT and δ-KO mouse forebrain membranes by GABA*,* GAA*,* γ-GBA*,* β-GPA*,* GES.* (**A**) Displacement curves, with specific binding normalized to protein concentrations show a consistent ~ 50% reduction of 5 nM [^3^H]muscimol binding in δ-KO forebrains compared with WT. Half-maximal concentrations for displacement (IC_50_) were calculated and are shown in the figures with standard deviations (SD). Since the IC_50_ for γ-GBA is significantly higher in δ-KO forebrain membranes when compared to WT, we calculated the δ-specific γ-GBA IC_50_ component by subtracting δ-KO from WT values (WT-δKO). [^3^H]muscimol displacement requires significantly higher concentrations of γ-GBA in δ-KO brains (*p* = 0.0035) when compared to WT, without significant differences between WT and δ-KO brains in displacement potency for GAA (*p* = 0.38), GABA (*p* = 0.41), β-GPA (*p* = 0.48) and GES (*p* = 0.91) (WT, wild type; KO, knockout; data are expressed as mean ± standard deviation). (**B**) Bar graph illustrating the highly significant (*p* = 5 × 10^-18^) reduction of total [^3^H]muscimol binding in δ-KO forebrain

Despite the low abundance of δ-receptors in the forebrain (~ 5% of total GABARs), we observed a highly significant ~ 50% reduction in high-affinity [^3^H]muscimol binding sites in δ-KO brains (Fig. 4B). The origin of the residual 50% of high-affinity [^3^H]muscimol binding in the δ-KO forebrain homogenates is unclear, since it is absent in δ-KO forebrain autoradiographic sections [44] and lacks functional equivalents (see Discussion).

Interestingly, the residual high-affinity binding in δ-KO mouse brains did not differ significantly from WT for GAA, β-GPA, GES and GABA displacement (with IC_50_ values given in Fig. 4A). Thus, WT and δ-KO IC_50_s closely reflect the displacement sensitivity for δ-GABARs. In contrast, significantly higher concentrations of γ-GBA were required to displace (5 nM) [^3^H]muscimol from δ-KO forebrain membranes (WT IC_50_ = 8.5 ± 0.9 µM, δ-KO IC_50_ = 26 ± 5 µM, *p* = 0.0035**). This shows that γ-GBA displays increased δ-selectivity, with a δ-specific (WT minus δKO) γ-GBA displacement IC_50_ of 3.5 ± 1.2 µM (Fig. 4A, upper right panel).

## Discussion

The goal of this study was to identify the molecular targets of GABA structural analog guanidino compounds and test the hypothesis that high-affinity extrasynaptic δ-GABARs are their primary sites of actions (see Fig. 1A). To this end, we compared the effects of four GABA-mimetic compound on native receptors in wild-type (WT) and δ-subunit knockout (δ-KO) mouse cerebellar granule cells (CGCs) using whole-cell recordings and cumulative concentration–response curves (Figs. 2 and 3), and also performed [^3^H]muscimol radioligand displacement assays in the mouse forebrain (Fig. 4). We have previously shown that GAA, γ-GBA and GES are orthosteric GABA agonists [17] and in this study also included, β-GPA, best known for its ability to block CRT1, the creatine transporter (see below).

Because extrasynaptic δ-GABARs are highly sensitive to GABA and activated by low ambient extracellular (typically *≤* 1 µM), we anticipated that, in the absence of highly agonist-sensitive δ-GABARs, structural GABA mimetics, would activate GABAR in δ-KO CGCs only at much higher concentrations. Indeed, for all compounds tested (including GABA itself), δ-GABARs deficiency resulted in significantly higher EC_50_ values (see Fig. 2; Table 1). The potency loss is also evident in the original traces: currents evoked at threshold concentrations for β-GPA, γ-GBA and GES are absent or smaller in δ-KO CGCs, consistent with the loss of high-affinity δ-GABAR-mediated responses. The absence of δ-GABARs is further reflected in steeper Hill slopes for β-GPA, γ-GBA and GES – indicative of the loss of a high-affinity current component - although this reached statistical significance only for β-GPA (see Fig. 2B, C, D; Table 1). Nevertheless, given the ~ 1000-fold difference in sensitivity to extracellular (≤1 µM) versus synaptic (mM range) GABA, the magnitude of the potency shifts for all compounds were surprisingly small.

A plausible explanation for the surprisingly modest losses of agonist sensitivity and similar maximal evoked steady-state currents (Table 1) is that the absence of δ-GABARs triggers a homeostatic upregulation of fairly agonist-sensitive α6β-containing GABAR composed of only α6 and β subunits. As previously reported, 24% of all GABARs were estimated to be composed of only α6 and β subunits in the δ-KO cerebellum, which is close to the estimated total number of δ-GABARs in WT cerebellar granule cells [31] - suggesting that α6β receptors may essentially substitute for the loss of extrasynaptic α6βδ in δ-KO CGCs. Under our conditions, with continuous agonist exposure, abundant synaptic receptors are expected to be fully desensitized and thus unlikely to contribute meaningfully to GABA-evoked currents. Our findings therefore support the interpretation that potency differences between WT and δ-KO CGCs reflect the smaller sensitivity gap between extrasynaptic α6βδ and α6β GABARs (with α6β GABARs replacing high-affinity α6βδ GABARs in δ-KO CGCs), rather than the expected ~ 1000-fold difference between α6βδ and synaptic αβγ GABARs. This interpretation is consistent with reports that recombinantly expressed GABARs composed solely of α and β subunits generally show much higher GABA/agonist sensitivity when compared to synaptic γ2-GABARs [32], although still significantly lower than δ-GABARs [33, 34].

To complement our electrophysiological data findings in CGCs, we performed [^3^H]muscimol displacement assays. Muscimol is a high-affinity GABA analog and mimetic that has been used for decades as a specific general GABAR ligand [45]. Both muscimol and THIP are reported to be highly selective for δ-GABARs [34, 36] and their high potency for δ-GABARs provides the molecular basis for the pronounced behavioral insensitivity of δ-KO and α4-KO mice to THIP [46, 47] and muscimol [47]. Notably, autoradiography studies showed an essentially complete loss of high-affinity (6 nM) [^3^H]muscimol binding in the δ-KO forebrain [44], in contrast to our findings (Fig. 4), which show that ~ 50% of 5 nM [^3^H]muscimol binding remains in the δ-KO in forebrain homogenates. The nature of this residual high-affinity binding in δ-KO forebrain homogenates is uncertain, but has also been observed in homogenates of recombinantly expressed γ2-GABARs, whose [^3^H]muscimol binding affinities are between 1 and 10 nM, whereas functional (EC_50_) muscimol responses that require muscimol concentrations ~ 1000-fold higher (i.e. 1–10 µM) [48]. This contrasts sharply with functional muscimol δ-GABA responses with an EC_50_ of ~ 1 nM for α4β3δ receptors [36]. Such high-affinity [^3^H]muscimol binding to non-δ GABARs in homogenates has therefore been proposed to represent non-functional desensitized synaptic GABAR [49] or artefacts arising from homogenization or freezing [36, 50].

Since the residual high-affinity [^3^H]muscimol binding in the δ-KO forebrain lacks any evidence for functional significance, it seems prudent to focus on the ~ 50% [^3^H]muscimol displacement component in the WT forebrain, that is lacking in the δ-KO forebrain. Interestingly, we obtained very similar IC_50_ values for [^3^H]muscimol displacement in both WT and δ-KO for GABA, GAA, GES, and β-GPA (except for γ-GBA). This indicates that both the δ-dependent and the residual non-δ-dependent components show very similar half maximal IC_50_ [^3^H]muscimol displacement (see Fig. 4A). Notably, these IC_50_ values align closely with our functional EC_50_ values supporting the interpretation that both the cumulated concentration-response electrophysiology and high-affinity (5 nM) [^3^H]muscimol binding assays primarily reflect high-affinity extrasynaptic GABARs. Binding assays often yield higher apparent potencies than functional measurements, but in our case IC₅₀ values for [³H]muscimol displacement were only modestly lower—largest for GABA at just over 10-fold—than EC_50_ values from CGC recordings. This small difference may reflect that both assays were performed at room temperature rather than on ice, the high agonist sensitivity of cerebellar α6βδ-GABARs compared with α4-containing receptors, and the fact that we did not calculate K_i_ values (expected to be lower) using the Cheng–Prusoff equation, as the Kᴅ for [³H]muscimol at α4βδ receptors is unknown.

The combined evidence that the four guanidino compounds studied here and elsewhere [17, 37] are (1) close structural GABA analogs (Fig. 1A), (2) activate functional GABA-like currents in CGCs, (3) which are completely blocked by the specific blocker gabazine (SR55731) (Fig. 2) and (4) completely displace [^3^H]muscimol at relevant and comparable concentrations (Fig. 4), strongly suggests that GAA, β-GPA, GES and γ-GBA are GABA mimetics acting on orthosteric GABA sites in GABARs (Fig. 1C). From a simple structure–activity perspective, the four guanidino compounds tested here preserve the core electrostatic and spatial features of GABA: a terminal positively charged group (either a primary amine, as in GABA, or a protonated amidino/guanidino group) paired with a terminal negatively charged group (a carboxylate in GABA, or, in the case of GES, a sulfonate), separated by an unbranched, flexible 2–4 atom aliphatic linker (Fig. 1A). This supports two key inferences. First, a negatively charged sulfonyl group can serve as an effective bioisostere for the GABA carboxylate at the orthosteric site, as also seen with homotaurine, which is structurally identical to GABA except for containing a sulfonate instead of a carboxylate group, a potent orthosteric GABA mimetic [37]. Second, replacement of the protonated terminal –NH_3_^+^ group in GABA with a protonated guanidino/amidino moiety (GAA, β-GPA, γ-GBA) maintains high apparent affinity and efficacy. Together, these observations argue that the orthosteric GABA pocket primarily recognizes the correct charge separation and linker geometry, rather than strictly requiring a primary amine on one end and a carboxylate on the other.

β-GPA is a high-affinity substrate for the creatine transporter, with an EC₅₀ of ~ 13 µM.- considerably lower than that of creatine itself (EC_50_ of ~ 35 µM) [20] - and under investigation as competitive creatine transport blocker cancer drug [23]. In this study, β-GPA displayed even higher potency on GABARs, activating tonic CGC currents (EC₅₀ ~3 µM, Figs. 2 and 3) and displacing [³H]muscimol in the forebrain (IC₅₀ ~4 µM. Figure 4). As a highly effective transporter substrate, β-GPA may gain access to the brain through the creatine transporter expressed at the blood-brain barrier, similar to the mechanism by which creatine enters the CNS in deficiency syndromes. Such transporter-mediated uptake could allow β-GPA to interfere with GABAergic neurotransmission centrally. Even if brain penetration were limited, β-GPA and other GCs could act on peripheral GABARs, including those on immune cells, potentially mediating anti-inflammatory effects akin to effects proposed for taurine and homotaurine [37].

δ-Selectivity of guanidino compounds may help explain the paradoxical hyperexcitability phenotype seen in GAMT deficiency, where the resulting massive increase in GAA (including in the brain), might activate GABARs at disease relevant concentrations [17]. Activation of δ-GABARs on inhibitory interneurons can reduce their firing, leading to disinhibition of downstream excitatory neurons - a key mechanism by which activation of tonic inhibition by GABA agonists, or δ-subunit gain of function mutations, can produce network-level excitatory effects, despite acting through inhibitory receptors [51, 52].

Overall, our data support the notion that structural GABA analogs and mimetics exhibit much higher affinity for extrasynaptic α4/6βδ GABARs – and, to a somewhat lesser extent, also for extrasynaptic αβ receptors - compared to synaptic γ2-GABAR. In δ-KO GCGs, the modest reduction in agonist sensitivity and preservation of tonic currents are consistent with homeostatic upregulation of high-affinity α6β receptors. These findings reinforce the view that higher GABA agonist affinity for extrasynaptic receptors is a common feature of orthosteric GABAR ligands, including guanidino compound GABA structural analogs like GAA, β-GPA, GES and γ-GBA.

## Data Availability

The data that support the findings of this study are available from authors upon reasonable request.

## Abbreviations

AGAT: Arginine: glycine amidinotransferase (EC 2.1.4.1)
GAMT: Guanidinoacetate N-methyltransferase (EC 2.1.1.2.)
CGC: Cerebellar granule cell
aCSF: artificial cerebrospinal fluid
GABA: γ-aminobutyrate
GABAR: γ-aminobutyrate type A receptor
δ-GABAR: extrasynaptic δ subunit-containing GABARs
γ2-GABAR: synaptic γ2 subunit-containing GABARs
δ-KO mice: δ-GABAR subunit deficient mice
GAA: Guanidinoacetate
GES: Guanidinoethanesulfonate
γ-GBA: γ-guanidinobutyrate
β-GPA: β-guanidinopropionate
α-K-δ-GVA: α-Keto-δ-guanidinovalerate
EC_50_: Half maximal effective concentration
IC_50_: Half maximal inhibitory concentration
I_max_: Maximal current at saturating agonist concentration
GCs: Guanidino compounds
